# The development of an integrated neighborhood approach for health promotion and prevention: a qualitative exploration of stakeholders’ views

**DOI:** 10.1186/s12961-023-01077-4

**Published:** 2023-11-28

**Authors:** Anniek Bosdijk, Anna Petra Nieboer, Jane Murray Cramm

**Affiliations:** Department of Socio-Medical Sciences, Erasmus School of Health Policy and Management, Erasmus University Rotterdam, P.O. Box 1738, 3000 DR Rotterdam, The Netherlands

**Keywords:** Integrated neighborhood approach, Health and social care, Health promotion and prevention, Interprofessional collaboration, The Netherlands

## Abstract

**Background:**

Although the promise of integrated neighborhood approaches, including the essential roles of communities and collaboration between the medical and social domains, has been widely acknowledged, the realization of such approaches in practice often remains difficult. To gain insight into the development of integrated neighborhood approaches, this case study describes the experiences of stakeholders involved in such an approach for health promotion and prevention in Rotterdam.

**Methods:**

Interviews with 18 stakeholders (including health and social care professionals, health insurance employees, and policymakers) were conducted, and stakeholders’ statements were analyzed thematically.

**Results:**

The results reveal a lack of alignment among the professional, organizational, and system levels. Elements needed for collaboration between health and social care professionals are not supported at the organizational and system levels. The lack of integration at the policy and organizational levels encourages competition and self-interest instead of stimulating collaboration.

**Conclusions:**

Intersectoral collaboration and coordination must take place not only between professionals, but also at the organizational and policy levels. As long as integration at the organizational and system levels is lacking, professionals’ ability to collaborate and provide coordinated support to neighborhood residents will be compromised.

## Introduction

Integrated neighborhood approaches (also called community-based or network approaches and health–social care partnerships) are increasingly advocated as means to prevent health declines, chronic disease onset, and increases in healthcare costs [1–4]. They consist of municipalities’ collaboration with health and social care providers to integrate available resources and increase responsiveness to citizens’ needs [3, 5–7]. Thereby, they are also expected to better serve more vulnerable citizens and thus contribute to the reduction of health inequalities [8, 9] Due to the promise that they hold as solutions for current and future healthcare challenges, these approaches are widely supported and have been tested in various countries. Despite agreement about their importance for the improvement of health promotion and prevention among citizens, their implementation in practice often remains complex, and results regarding their effectiveness are mixed [10–12]. Various obstacles at the policy (e.g., inadequate payment mechanisms), organizational (e.g., lack of commitment), and interpersonal (e.g., poor care coordination) levels hamper adequate integration and collaboration, and have prevented the implementation of integrated neighborhood approaches in some cases [13–18]. A better understanding of current barriers and opportunities, gained through the examination of cases in practice, is needed for the development of successful integrated neighborhood approaches.

### The Rotterdam case

This article describes the development of an integrated neighborhood approach in Rotterdam, the second largest city in the Netherlands. Rotterdam is multicultural, with more than half of its > 650,000 inhabitants having migration backgrounds with people of Surinamese, Turkish, Moroccan, and Antillean origin forming the largest minority groups [19]. In 2022, Rotterdam was declared “the unhealthiest city of the Netherlands” due to the physical living environment [20]. Rotterdam’s inhabitants are also unhealthy relative to the rest of the Dutch population [21], and large health disparities exist in the city. The average life expectancy in perceived good health, for instance, ranges from 51.3 years in the Feijenoord District to 63.2 years in Hillegersberg-Schiebroek [22]. Important causes of such disparity are differences in education, income, work, and unhealthy behaviors [21]. These increasing health problems, accompanied by major differences among areas and societal groups, indicate the need for an integrated neighborhood approach in Rotterdam.

In early 2022, the municipality of Rotterdam and a health insurer initiated a project to develop a “preventive neighborhood infrastructure” in three neighborhoods. The Rotterdam care and support system is threatened, as the number of people in the city with (chronic) illnesses and conditions is increasing rapidly and preventive (lifestyle) interventions and social services are insufficiently used. The project focuses on health promotion and prevention and aims to encourage a shift away from the medical domain. Stronger collaboration and linkages between primary care and social services in neighborhoods are assumed to be essential for the proper provision of care and support at the right place and time. The project initiators stated that such collaboration had not been established sufficiently in Rotterdam and sought to remedy the situation together with various parties in the pilot neighborhoods, including social care organizations, sports providers, and general practitioner (GP) practices. When proven successful, the approach is intended to be scaled up with implementation in other parts of the city.

### Aim

The importance of integrated neighborhood approaches, including the essential roles of communities and collaboration between the medical and social domains, has been acknowledged widely. Hartgerink and colleagues [23], for instance, argued that interprofessional collaboration is the main factor influencing the effectiveness of integrated care delivery. Lette et al. [24] also concluded that the improvement of integrated care begins with the improvement of interprofessional collaboration. The implementation of integrated neighborhood strategies in policy and practice, however, often proves to be complex. An important step in the development of a successful intervention is the identification and understanding of the prevailing problem and its causes [25]. This case study describes the views of stakeholders involved in the development of an integrated neighborhood approach in Rotterdam. Our main objective was to explore the current barriers to and opportunities for increased collaboration between the medical and social domains in the pilot neighborhoods, with a greater focus on health promotion and prevention. The lessons learned from the Rotterdam case may inform the future development of similar initiatives elsewhere.

### Theoretical framework

Several conceptual frameworks explain key elements of successful integrated care approaches [26]. A widely used model for the organization of integrated chronic care is the chronic care model (CCM), which attributes improvements in functional and clinical outcomes to productive interactions between informed, activated citizens and proactive practice teams [27]. This model, however, has been criticized for its narrow focus on clinically oriented systems and individual health outcomes [28]. Barr et al. [28] developed the expanded CCM, which encompasses health outcomes and the well-being of citizens, communities, and populations.

Most initiatives for health promotion and disease prevention are implemented at the community level. The expanded CCM places greater emphasis on the important role of formal and informal community support [28]. It holds that improvements in public health and individual clinical outcomes are the results of productive interaction and relationships among individuals, community members/groups, healthcare professionals, and organizations. It identifies several important components at the community level: (a) *building healthy public policy* to improve population health; (b) *creating supportive environments* to promote safe, stimulating, satisfying, and enjoyable living and working conditions; and (c) *strengthening community action* to empower communities to set priorities and achieve goals for health promotion. Four additional components are identified within and between the health system and community: (a) *self-management/personal skills development* for health and well-being; (b) *delivery system design/health services reorientation* to provide more holistic care and support to individuals and communities that focuses not only on clinical and curative services, but also takes broader social, political, economic, and environmental aspects into consideration; (c) *decision support* via guidelines for disease management and decision making to benefit citizens’ health and well-being; and (d) the accessibility of *information systems* containing health-related data and relevant community information (e.g., demographic, social, and economic trends) to all parties involved [28].

The expanded CCM provides a suitable framework for the examination of the integrated neighborhood approach in Rotterdam, which aims to increase the integration and coordination of social and care services with a greater focus on health promotion and prevention. This model, however, does not explicitly identify the levels at which care integration must be achieved or the interplay among them. The rainbow model of Valentijn and colleagues [29], which details the need for integration at different system levels for continuous, comprehensive, and coordinated care delivery, is thus useful. For instance, this model holds that *clinical integration* is required at the microlevel; individual care services should be person focused and coordinated across times, places, and disciplines [29]. At the mesolevel, *organizational integration* and *professional integration* are needed; organizations must collaborate and coordinate to provide comprehensive services to certain populations, and professionals should partner within and between organizations, taking shared responsibility for the delivery of coordinated support. At the macrolevel, *system integration* is desired. Rules and policies within a system must be aligned and serve the central purpose of meeting people’s needs. The rainbow framework also has two integrative dimensions that span the three levels: *functional integration* (the coordination of support functions and activities, such as information systems and financial management) and *normative integration* (the establishment of a common frame of reference, with, for example, a shared mission and joint values).

The two models highlight important aspects of the organization of integrated neighborhood approaches from slightly different, complementary perspectives. Their combined use can provide better insight into why an integrated neighborhood approach works or does not, and at which levels and/or in which aspects change is needed to achieve better outcomes. The focus of the expanded CCM on health promotion and prevention, with the community and local practice teams playing central supporting roles, is in line with the main goals of the Rotterdam initiative examined here. In addition, the forms of integration distinguished by Valentijn et al. [29] are used to determine at which levels barriers and opportunities are located.

## Methods

### Design and participants

This qualitative case study is part of a larger action research project for the development of an integrated neighborhood approach for health promotion and prevention in Rotterdam. Seventeen semi-structed interviews with stakeholders were conducted in August–December 2022. Eligible stakeholders were involved with the project and/or employed in the medical or social domain in at least one of the three pilot neighborhoods. The municipality recommended the participation of several stakeholders. Snowball sampling was used to include other relevant stakeholders. Sampling stopped when data saturation was reached, with no new barrier to or opportunity for intersectoral collaboration mentioned in additional interviews. Eighteen stakeholders operating in various sectors at different levels, ranging from those working in general practices and welfare organizations to health insurance employees and policymakers, participated in the study. Table 1 provides an overview of the participants.

**Table 1:** Overview of Stakeholders

	Gender	Age	Background
Municipality	Woman	58	Policy officer
	Woman	46	Policy officer
	Woman	45	Department head
Health Insurer	Woman	55	Strategic advisor
	Man	62	Healthcare purchaser
Social domain	Woman	32	Community worker
	Woman	39	Community worker
	Man	45	Community worker
Medical domain	Woman	45	Neighborhood coordinator^a^ + practice nurse
	Woman	49	Neighborhood coordinator
	Woman	53	Neighborhood coordinator
	Woman	51	Neighborhood coordinator + GP practice manager
	Woman	46	Practice nurse
Other	Woman	50	Sports professional—project manager
	Man	33	Sports professional—consultant
	Woman	63	Healthcare and welfare consultant
	Man	60	Physical therapist
	Woman	50	Developer lifestyle intervention

^a^This function entails setting up partnerships between GPs and connecting them with neighborhood partners

### Data collection

According to stakeholders’ preference, the interviews (45–90 min) were conducted online (*n* = 7; via Microsoft Teams) and in person (*n* = 11; e.g., at GP practices, community centers, cafes, and the municipal office). The first author conducted all interviews. With the stakeholders’ consent, all interviews were audio recorded. The interviews were transcribed verbatim. Our aim was to explore current barriers to and opportunities for increased collaboration between the medical and social domains, with a greater focus on health promotion and prevention. An interview guide (Appendix 1) was developed. It included questions pertaining to the levels of the rainbow model, such as those about the influence of professionals, organizations, and rules and policies at the macrolevel. It also touched on elements that are important for the provision of integrated support, according to the literature (e.g., expanded CCM components such as the role of public policy and organization of delivery and information systems). The guide was adjusted slightly in some cases to match the stakeholders’ domain and level of operation. Briefly, the stakeholders were asked to describe their positions and the roles of prevention and collaboration with other domains, organizations, and professionals in their daily work activities. They were asked to speak about previous positive and negative experiences, obstacles, and desires for the future, and to identify (sensitive) topics or possible pitfalls that could contribute to the failure of the integrated neighborhood approach.

### Data analysis

The data were analyzed thematically using the six phases described by Braun and Clarke [30]. After becoming familiarized with the data, the first author performed initial coding inductively. The codes were sorted into potential themes, which were critically reviewed and defined. Examples of identified themes were trust, communication, professionals’ mentality, and (un)familiarity with neighborhood services. Coding was performed with the aid of the ATLAS.ti 22 for qualitative analysis. The coding scheme was discussed with and reviewed by the other two authors. Data collection and analysis were performed in Dutch to avoid loss of meaning. Stakeholders’ quotations were translated into English in the final phase of the research. Trustworthiness was improved by several measures. A summary of each interview was created and sent to the stakeholder to check whether s/he agreed with the researcher’s interpretations. Preliminary findings were also presented in a setting with several stakeholders present. After the presentation, the researcher asked whether the conclusions drawn matched the stakeholders’ personal experiences. The stakeholders answered in the affirmative.

### Ethical considerations

The ESHPM Research Ethics Review Committee at the Erasmus University of Rotterdam approved this study (ETH2122-0801). Before being interviewed, the stakeholders received and signed an informed consent form that contained information about the study, including its aims, the voluntariness of participation, guarantee of anonymity, and data management.

## Results

Stakeholders’ experiences with intersectoral collaboration and their perspectives on the integrated neighborhood approach in Rotterdam are described in this section. Experiences at the professional, organizational, and policy levels are described in the order stated.

### Professional level

At the professional level, *mutual trust*, *familiarity with the services available*, and *a collaborative mindset* were common themes for intersectoral collaboration in the neighborhood.

#### Mutual trust

In the Rotterdam neighborhoods, collaboration between professionals occurred primarily via client referral. The stakeholders mentioned that trust in each other and the other’s expertise was thus of great importance. Professionals wanted to be sure that their clients received appropriate support. In the absence of trust, they indicated that they tried to help their clients themselves, regardless of whether suitable support was available elsewhere.

The stakeholders identified various elements that contributed to or detracted from mutual trust. A key element was clear and timely communication, with openness and honesty about one’s interests and expectations, and clear arrangement making. They indicated that defective or incomplete communication undermined trust. For example, a community worker said that when she did not receive feedback from the receiving professional after referring a client, she assumed that the professional had done nothing with the case In Rotterdam, lack of communication is often cited as a reason for the difficulty of collaboration between GPs and social neighborhood teams, with GPs receiving too little feedback. A neighborhood coordinator/GP manager stated:

“A common counterargument frequently voiced by GPs is their perceived lack of feedback. Without this feedback loop, they remain unaware of developments and are less inclined to make referrals in the future.”

The stakeholders also noted that personal relationships were main contributors to mutual trust between professionals, explaining that in-person familiarity helped them to remember others’ professional focus and working method, which made collaboration and client referral more likely. A sports professional, for instance, mentioned that you would not be likely to refer a client when you had no idea with whom they will come into contact with.

The stakeholders preferred to get to know each other face to face and physically meet in the work environment, as this approach contributed to a better understanding of each other’s worlds. Some stakeholders felt that personal connection facilitated collaboration with professionals outside of their organizations. However, they noted that the building and maintenance of personal relationships required time and effort. In Rotterdam, this process is complicated by the large number and frequent changes of professionals and organizations in neighborhoods, which make the development of personal relationships with relevant network partners a continuous task. A neighborhood coordinator brought up the municipality’s assignment of the welfare tender to one provider per neighborhood and retendering every 4 years:

“After four years, a new organization comes into play, often involving different individuals. This situation frequently triggers significant turmoil, leading people to eventually lose motivation. They cease investing additional effort, expressing sentiments like, “I won’t engage with this welfare organization anymore because it keeps changing with new faces and structures, requiring me to rebuild connections from scratch every time”.”

In June 2022, the municipality extended welfare assignments to up to 10 years to allow providers to build long-term relationships with residents, neighborhood network partners, and the municipality [31]. Several stakeholders mentioned this as a positive policy change.

Previous experiences with collaboration also influenced the degree of trust between professionals, with positive experiences strengthening mutual trust and negative experiences generating more hesitant attitudes about collaboration. A community worker spoke about the reputational damage caused by his predecessor’s poor communication:

“During certain meetings [with other neighborhood organizations], there is a noticeable sentiment, not necessarily openly opposed to [name of the welfare organization], but rather an undertone of “the welfare organization doesn’t follow through on its commitments.” This sentiment doesn’t prevail as the primary atmosphere, but when such a signal is heard, it can be frustrating, and in my view, unwarranted. It’s simply an impression that tends to linger, particularly if there was a past employee who occasionally missed meetings without adequate communication – such instances can contribute to the formation of such an image.”

Stakeholders indicated that like communication, personal relationships with shared positive previous experiences contribute to mutual trust. Conversely, professionals who have not gotten along in the past may be reluctant to collaborate.

#### Familiarity with the services available

Information about available neighborhood services is essential to provide appropriate and timely support. A professional who is unaware of certain opportunities for support, especially in other domains, obviously will not recommend them to his/her clients. A community worker mentioned that collaboration sometimes does not work, due to the lack of clarity and knowledge of whom to call. Several stakeholders noted the great need for a “social map” providing an overview of the neighborhood services available, but at the same time recognized the impossibility of properly creating and maintaining it. A neighborhood coordinator/GP manager stated:

“There’s mention of the social map, which is also accessible online. However, it can be frustrating when you locate your designated contact person, give them a call, only to discover that they are no longer in that position. I recall hearing my colleagues say, “It’s nearly impossible to maintain something like this entirely up-to-date.” This is because changes occur so frequently, and it’s simply unfeasible to keep track of them in real-time.”

The creation and maintenance of personal relationships is thus time consuming, given the many changes in neighborhood services and active professionals. Stakeholders noted that not all professionals could take this extra time, especially in the current context of labor shortages and increasing requests for help. A neighborhood coordinator, for instance, pointed out the importance of practice nurses taking the time required to become familiar with the neighborhood network for appropriate patient referral. However, most general practices have no budget or way to fund such networking.

In contrast, networking—specifically the creation of network maps and connecting of partners—is the core task of some professionals in the neighborhoods, including those from welfare and GP organizations. Organizations can approach them to obtain information about where to go in specific cases. However, the stakeholders noted that the large number of networker-like actors from different organizations and with different perspectives creates confusion. A neighborhood coordinator explained:

“Sometimes, I find myself in a meeting and wonder, “Wow, do you have that too?” Within the municipality, there’s a neighborhood manager for each neighborhood, a neighborhood support worker, a neighborhood concierge, and a neighborhood networker, and I’m still not entirely clear on their specific roles. [...] It’s been three and a half years, I'm still uncovering new things. But if I’m still not fully informed, how can a GP be expected to know?”

#### A collaborative mindset

Successful intersectoral collaboration also requires professionals to have a collaborative mindset. Many described one element of this mindset as a ‘broad perspective’ with an understanding of the importance of other disciplines’ contributions. For example, a sports professional said that it helped when professionals in other domains were also sports minded and viewed exercise as a possible part of the solutions to their clients’ problems. Stakeholders felt that professionals should be able to recognize when services from another provider are more appropriate for a client at a certain time and place and (temporarily) step aside. A healthcare consultant stated:

“We should adopt the perspective that everyone plays a distinct role, and each role is equally important when it comes to patient care because each person brings something unique to the table. Collectively, we share a mission, I would say, to enhance the quality of life for that individual. I’ve often compared it to the functioning of a nursing home, where you have individuals responsible for the cleaning, managing invoices, and providing direct care. If the cleaners no longer do anything or the invoices are no longer sent, the house collapses too.”

The stakeholders noted the need for professionals to understand the importance of collaboration and to view it as an integral part of their jobs, rather than as an extra element on top of their existing work activities. A community worker explained that professionals make time for collaboration when they perceive it as important, but are quick to drop it when they view it as something extra.

Besides a positive and willing attitude, stakeholders mentioned other essential elements for collaboration, such as neighborhood partners’ having shared definitions, visions, and goals. This normative integration is of great importance for the development of an integrated neighborhood approach, and there is room for improvement on this matter in Rotterdam. In general, the stakeholders perceived neighborhood connections and health prevention as important, but had different perceptions of what prevention entailed. One stakeholder felt that the concept morally dictates what a person can and cannot do, and another preferred to talk about “health” instead of prevention. Others defined the concept as “preventing the occurrence of” and argued that this does not occur in general practice, as people visit GPs when problems are already present. In turn, these views of prevention do not correspond with the preventive efforts of the municipality of Rotterdam, which has chosen to build preventative neighborhood networks using two existing interventions in which GPs play central, signaling roles and refer clients to the social domain. One of these interventions is the combined lifestyle intervention, which aims to improve the lifestyles of people with overweight and obesity. A lifestyle intervention-developer stated:

“I appreciate the effort to assess what’s necessary for enhancing prevention. However, If I were to express my cynical perspective, it appears that we aren’t genuinely engaged in prevention efforts. Instead, we seem to be addressing individuals who already grappling with health issues, such as having a BMI over twenty-five with a comorbidity or a BMI over thirty, almost guaranteeing future comorbidities. If the true aim is to focus on prevention, it might already be too late by the time a combined lifestyle intervention is initiated.”

For a collaborative mindset, stakeholders also noted the need for clear agreements and division of tasks between collaborating parties. Professionals must clearly understand others’ roles, know to whom they can turn for specific services, and avoid encroaching on others’ scopes and feeling the need to compete, which hampers the development of a collaborative mindset. Moreover, the stakeholders felt that professionals’ openness about their expectations (and ability to meet them) and personal/organizational interests was required. The setting of inappropriate expectations and subsequent failure to meet them create a negative collaboration experience, which reduces mutual trust, described previously in this paper.

### Organizational level

In practice, effective intersectoral collaboration requires proper support at the organizational level. The stakeholders’ statements indicated that professionals in Rotterdam are not always given sufficient time and opportunity to get to know each other and become familiar with the services available, and to build relationships of trust. The stakeholders mentioned *conflicting assignments*, *competition*, and *incompatible information systems* as organizational elements that hinder intersectoral collaboration.

#### Conflicting assignments

Organizational interests can hamper collaboration; in Rotterdam, each organization in the medical and social domains is tasked with completing its own (often temporary) assignment from the municipality, with specific requirements and objectives set for associated funding. Collaboration is often not a part of these assignments or an element of performance measurement, and thus perceived as something “extra.” Especially during periods of personnel shortages or work pressure, organizational goals are often given priority. In line with other stakeholders’ comments, a community worker stated:

“When you’re short on time to complete neighborhood rounds, you’re less likely to dedicate an hour to participate in a network meeting, for example.”

Many stakeholders perceived the achievement of key performance indicators (KPIs; quantitative measures of organizational performance against set objectives) to be important, but also to hinder collaboration. They stated that organizations must sometimes meet certain objectives, such as helping a set minimum number of clients, which makes transfer to neighborhood partners’ perhaps more suitable support services less attractive. A sports professional said:

“When we all have KPIs, such as targets for the number of individuals we need to support, there are instances where you might refrain from making referrals, because those are your clients, your goals.”

A more qualitative approach was suggested to stimulate organizations’ joint achievements. For example, cases in which residents who have received proper support from a suitable service through collaboration could be showcased. Stakeholders noted that the focus must be on the meeting of collective goals, accompanied by (joint) financial incentives, rather than on the interests of individual organizations, but that functional (e.g., finance coordination) and normative (e.g., shared mission and values) integration was lacking. The stakeholders perceived that individual organizational assignments and goals at the organizational level are at odds with identified needs at the professional level, promoting self-interest rather than a collaborative mindset.

#### Competition

Competition between organizations (within and among domains) was also mentioned as a barrier to collaboration. Reasons given for competition included clients, task division in the neighborhood, associated financial resources, and organizations’ assertion of their right to exist. For example, some sports and welfare organizations compete, with tension over ownership prevailing; some welfare organizations receive funding to organize exercise activities, such as walking groups, which some sports providers perceive as encroaching on “their business.” A sports professional stated:

“The municipal budget is a finite resource, and when one organization secures funding, it often means another misses out. In our case, we argue that the budget rightfully belongs to Sports. However, Welfare contends that Sport doesn’t effectively serve their clients, although there is still a lingering notion that “maybe we can do it ourselves.”

The stakeholders argued that collaboration at the professional level requires shared goals, mutual agreement, and clear task division, and that organizations’ perception of each other as competitors leads to distrust between professionals.

#### Incompatible information systems

Good communication and feedback, essential for trust and collaboration between professionals, can be facilitated by working in the same digital information systems (an element of functional integration), which in turn makes client referral much easier. A physiotherapist, for example, stated that he had been much busier since joining the same digital system as the GP. Other professionals working in the same GP system also had mainly positive feedback about this development and experience, which resulted in easier and more frequent communication and collaboration.

Conversely, the stakeholders cited the lack of functional integration resulting from working in different information systems as an important barrier to collaboration. They found working and communicating through different channels to be confusing and inconvenient, and to require extra time and effort from professionals. For example, the regularly mentioned difficulty of collaboration between GPs and neighborhood teams (who work on behalf of the municipality) in Rotterdam often stemmed from the use of different digital information systems. GPs must refer patients using separate registration forms, and receive little information and feedback about referral progress. A practice nurse stated:

“I prefer directing individuals to the neighborhood team, but the process is somewhat challenging. It involves filling out their application form, which can be frustrating, time-consuming, and impractical. When I attempt to complete it on the computer, I sometimes make errors or encounter technical glitches, resulting in a blank page. It’s unclear whether it’s my mistake or if there are occasional system issues. Additionally, there are occasions when we provide a detailed and compelling referral, send it off, and then it seems to disappear into the void, with minimal follow-up or action taken.”

### System/policy level

Various system-level elements of current health insurance and municipal policies were named as barriers to intersectoral collaboration. For instance, the municipality of Rotterdam is a large organization consisting of various clusters, boards, departments, and teams with employees working on various themes. Municipal employees and professionals from other domains noted the lack of internal coordination and cohesion within the municipality. Many described the departments as islands that are not aware of the activities and projects of fellow policy officers and do not collaborate with them. For example, a municipal employee noted that colleagues are sometimes doing essentially the same work without realizing it. This incoherence within the municipality has several consequences, reflected in the three main barriers that emerged from the discussions with stakeholders, namely *similar assignments given to multiple parties*, *overtasking of professionals*, and a *skeptical attitude* about new initiatives.

#### Similar assignments given to multiple parties

In Rotterdam, similar assignments are given to multiple neighborhood organizations from different angles. For example, similar neighborhood network–like entities have been developed by organizations focused on youth, older adults, welfare, and GPs. The stakeholders mentioned the overlapping roles of neighborhood directors, brokers, coordinators, and managers, among others. Most professionals lacked clarity about what given functions entailed. They stated that the current municipal organization, rather than facilitating such familiarity, created more chaos and uncertainty at the professional level. A municipal employee said:

“We find ourselves unintentionally assigning responsibility for organizing specific networks to six different parties, without realizing that the same individuals are involved in multiple networks. Consequently, we end up with essentially the same group of people gathered around six different tables. The only difference might be that one table focuses on older adults while another focuses on youth, but the participants remain largely identical. This also highlights an internal challenge for us—how do we avoid duplicating efforts and providing similar directives to six different parties? If chaos is what we’re aiming for, this approach certainly achieves it.”

#### Overtasking of professionals

Various stakeholders stated that the same professionals were always called upon, despite the existence of many neighborhood consultation structures, initiatives, and pilot projects, including several initiated by the municipality and/or health insurer. The lack of cohesion at the policy level results in the allocation of several, sometimes similar, tasks to the same neighborhood professionals by employees in different policy areas and departments. A municipal employee stated:

“I found it rather shocking when my colleague shared her analysis of all the demands placed on professionals by the municipality. Some individuals are members of up to 21 associations or teams within their local area, all connected to the municipality of Rotterdam. When we discuss efficiency and strategic organization, it’s evident that as the municipality of Rotterdam, we might not be handling this very effectively. Overloading professionals in this manner undoubtedly saps their enthusiasm.”

Some stakeholders involved in projects other than their main foci noted that attending meetings, contributing ideas, and providing input requires professionals’ time and effort outside of their regular work activities. Although many stakeholders consider their involvement in such developments to be important, it limits their ability to provide support to neighborhood residents. A sports professional said:

“I’m partly funded by the municipality, so I do feel an obligation to participate in interviews like this. However, on the other hand, I can’t help but think, “During this time, I could have helped people,” and I genuinely prefer doing that because they need my help more than you do for this interview.”

#### Skepticism

In line with the previous issue, some stakeholders did not feel heard by the municipality; they had participated in various projects, but do not feel that anything had been done with their input. A sports consultant, for instance, mentioned that he had voiced his opinion many times, but that nothing had been done in response. Several stakeholders stated that projects stop when the funding runs out, and all that has been achieved disappears. Despite the widely shared recognition of the importance of more collaboration and a focus on prevention, several stakeholders reacted to the prospect of new project development with skepticism and reservations. A neighborhood manager noted:

“In the neighborhood, I sometimes notice instances of overlapping initiatives or situations where there’s an excess of activities. Various projects, often initiated by the municipality, kick off as small endeavors with initial funding, but after the funds run out, it subsides again.”

The professional, organizational and policy levels described are interconnected and affect each other. In Rotterdam, the identified barriers at the organizational and policy levels hinder the observed opportunities at the professional level. First, incompatible information systems at the organizational level complicate communication needed at the professional level to establish mutual trust. Second, professionals need time and space to become acquainted with neighborhood services and partners. Similar assignments given to multiple neighborhood parties by the policy level confuses this process, and overtasking professionals limits their ability to develop and maintain personal relationships. Third, conflicting assignments at the organizational level make collaboration an additional task instead of an integral part of professionals’ jobs. Together with competition at the organizational level, the latter hampers a collaborative mindset for professionals.

In sum, in Rotterdam professionals are expected to collaborate, which requires time and investing in each other, familiarity with the neighborhood and the right mentality. Instead of facilitating this, however, the organizational and policy levels seem to hinder this, by overloading professionals, imposing multiple conflicting tasks and creating chaos and unclarity in the neighborhood.

**Fig. 1:**
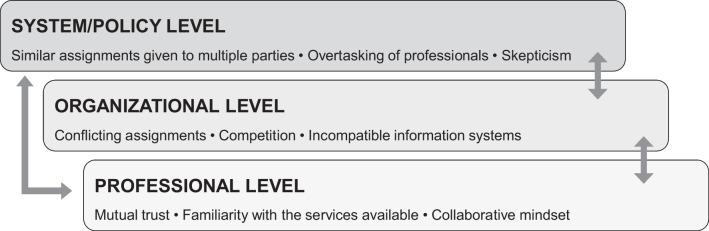
Overview of barriers and opportunities in Rotterdam’s integrated neighborhood approach

Figure 1 provides an overview of the levels involved in the integrated neighborhood approach in Rotterdam, with identified barriers and opportunities for intersectoral collaboration.

## Discussion

This study was conducted to improve the development of an integrated neighborhood approach for health promotion and prevention by exploring stakeholders’ views on current barriers to and opportunities for collaboration between the medical and social domains in Rotterdam neighborhoods. The findings show that alignment among the professional, organizational, and system levels is currently lacking. Professionals indicated that their need for more collaboration, entailing mutual trust, familiarity with the services available, and a collaborative mindset, is not supported at the organizational and system levels. On the contrary, the lack of integration at these levels encourages competition and self-interest, rather than stimulating collaboration.

### The importance of interprofessional collaboration

Interprofessional collaboration is essential for the delivery of integrated care [23, 24]. The stakeholders mentioned that mutual trust, created and strengthened by communication, positive previous experiences, and a collaborative and respectful attitude toward other professionals, was important for such collaboration. Consistent with these findings, professionals’ cognition and behavior [23]; relationships of trust, understanding, and communication [24]; the pursuit of common goals and mutual respect [32]; and operation with a shared culture and vision [33] have been identified as important prerequisites for interprofessional collaboration [34]. These professional-level requirements are not sufficiently present in the pilot neighborhoods in Rotterdam.

The facilitation of interprofessional collaboration is thus of great importance for the development of a successful integrated neighborhood approach. Professionals need to be familiar with each other and the services available. Neighborhood social maps are desired, but keeping them up to date is an ongoing task due to the wide range of services and frequent changes of organizations and professionals in the Rotterdam neighborhoods. Efforts to address this issue involve the creation of new neighborhood coordination and networking positions, with professionals instructed to map out the services available, make neighborhood plans, connect professionals, and/or set up networks. Our findings, however, show that such efforts paradoxically hinder collaboration, creating chaos and uncertainty instead of a better overview due to the allocation of similar tasks to a large number of professionals from various sectors and perspectives. These findings are in line with the argument that the existence of multiple overlapping network structures complicates prioritization and action taking [35], and the emphasis of the importance of clear roles and allocation of network management [36, 37].

In Rotterdam, solutions to professionals’ lack of familiarity with each other and with neighborhood services have been sought mainly at the professional level. Our results, however, indicate that changes at the organizational and system levels should also be made to facilitate interprofessional collaboration and thereby achieve the desired integration in practice. Similarly, Hartgerink et al. [23] highlighted the importance of a supportive organizational context, including integrated information and communication systems. Wilderink and colleagues [38] identified multilevel (strategic, tactical, and operational) initiative support with vertical communication and the embedding of the approach in organizations’ policies and processes (e.g., financing structure, daily professional and organizational tasks) as key elements of the success of an integrated community-based approach in Zwolle, the Netherlands. The framework of Cheng and Catallo [33] includes dedicated funding and resources as factors enabling collaborative health and social care integration, as well as several contextual factors beyond collaborating organizations’ immediate control that can act as both barriers to and enablers of integration (i.e., policy, sector funding models, governance structures, and geographical setting).

### The need for coherence

The lack of coherence in the municipality of Rotterdam and fragmentation at the policy level identified in this study trickled down to the organizational and professional levels. The situation in which assignments, funding, and positions from separate pillars are associated with their own goals and have little connection to other sectors does not facilitate service integration at the professional level. Similarly, Van Dijk et al. [7] identified conflicting organizational interests and inadequate financial incentives as challenges to an integrated neighborhood approach for community-dwelling older people in Rotterdam, concluding that meso- and macrolevel contexts did not support community workers’ achievement of integration. The improvement of cohesion and coordination at the policy level would affect the organizational and professional levels, and should facilitate intersectoral collaboration in practice.

### Theoretical considerations

Our study shows that the development of an integrated neighborhood approach requires substantive elements of the expanded CCM and the rainbow model. The stakeholders’ perspectives and the resulting recommendations reflect these elements. The Rotterdam case, in which the practical applicability of the models was explored, contributes to further theorizing. To achieve professional integration with the objective of health promotion and prevention in Rotterdam’s neighborhoods, changes in the health system and broader community from the expanded CCM should be made. For instance, public policy that promotes intersectoral collaboration and reorients services by bundling organizations’ assignments, with appropriate joint and structural funding and common performance indicators, should be developed. Such policy may reduce professionals’ inclination to compete and facilitate their prioritization of the pursuit of shared goals. The results of this study indicate that lack of normative and functional integration from the rainbow model, as well as the lack of system integration, at the macrolevel, makes it difficult to expect professionals to pursue shared missions and coordinate support services in practice. Setting the right example at the policy level, with shared policies, collaboration, coordination, and integration among involved sectors, would facilitate similar implementation at the lower levels, including organizational and professional integration at the mesolevel.

### Limitations

Some limitations of this study should be mentioned. First, the insights obtained in this study are context specific and cannot simply be transferred to all neighborhoods. Nevertheless, the findings may inform the development of similar initiatives for contexts in which the same barriers and/or opportunities exist. Second, the perspectives of neighborhood residents were not explicitly considered in this study. Indeed, residents have not yet been directly involved in the development of the integrated neighborhood approach. De Jong and colleagues [39, 40] argued that citizen participation, in addition to intersectoral collaboration, in community health-promotion programs is important. Their research shows that professionals’ and citizens’ perspectives on elements that are relevant for health and healthy behaviors differ [39]. Other researchers have also emphasized the importance of collaboration with citizens on integrated care initiatives and health promotion and prevention programs [38, 41, 42]. Thus, future research on the development of integrated neighborhood approaches should involve the exploration of citizens’ views and enhanced citizen participation to ensure that the efforts made match residents’ support needs.

## Conclusion

Interprofessional collaboration is essential for the development of an integrated neighborhood approach. In the case of Rotterdam, the desired intersectoral collaboration has been complicated by the lack of alignment among the professional, organizational, and system levels. Professionals’ needs, such as relationships of mutual trust, a collaborative mindset, and familiarity with the services available, are not supported at the organizational and policy levels. A lack of integration at the policy and organization levels encourages competition and self-interest instead of stimulating collaboration. As a result, health and social care professionals do not get the time and space needed to invest in each other and collaborate. As long as cross-domain integration at the organizational and system levels is lacking, professionals’ ability to collaborate and provide coordinated support to neighborhood residents will be compromised.

## Data Availability

The data used to support the findings of this study are available from the corresponding author upon reasonable request.

## Appendix 1

### Topic list for stakeholder interviews

**Table:**

Topic	Elements
Stakeholder	Background, function, organizational role, daily activities, demographic data (age, gender, residence)
Organization	Domain, activities, target group, expertise, current role in neighborhood/community
Neighborhood	Characteristics, residents, problems, organizations present, community, geographical setting
Integrated neighborhood approach	First thoughts and impressions
Prevention	Definition, importance, current role in organization/activities
Interprofessional, -organizational, and -sectoral collaboration in the neighborhood	Previous experiences, lessons learned, factors contributing to success and failure
Referral to other professionals/organizations	Information systems, monitoring/visibility results, trust, personal relationships
Barriers to collaboration in the neighborhood	Current obstacles, potential pitfalls, sensitive topics
Needs for improved collaboration	• System: healthy public policy, funding • Organization: vision and goals, means and resources, information systems, delivery system design, division of tasks/responsibilities • Professional: personal relationships, trust, skills, competencies
Integrated neighborhood approach development	Important roles, possible professional and organizational roles
